# Recruiting patients as partners in health research: a qualitative descriptive study

**DOI:** 10.1186/s40900-017-0067-x

**Published:** 2017-08-21

**Authors:** Lidewij Eva Vat, Devonne Ryan, Holly Etchegary

**Affiliations:** 1 Training and Capacity Lead, NL SUPPORT Unit, Memorial University of Newfoundland, Faculty of Medicine, Room 4M104, St. John’s, NF A1B 3V6 Canada; 2 Research Assistant/ PhD Candidate, Memorial University of Newfoundland, Clinical Epidemiology Unit, Faculty of Medicine, Suite 4M120, St. John’s, NF A1B 3V6 Canada; 3 Assistant professor/ Patient Engagement lead NL SUPPORT Unit, Memorial University of Newfoundland, Clinical Epidemiology Unit, Faculty of Medicine, Craig L. Dobbin Centre for Genetics, Room 4M220, St. John’s, NF A1B 3V6 Canada

**Keywords:** Patient engagement, Patient and public involvement, Health research, Recruitment, Stakeholder engagement

## Abstract

**Plain English summary:**

Increasingly, funders and researchers want to partner with patients in health research, but it can be challenging for researchers to find patient partners. More than taking part in research as participants, patient partners help design, carry out and manage research projects. The goal of this study was to describe ways that patient partners have been recruited by researchers and patient engagement leads (individuals within organizations responsible for promoting and supporting patients as research partners).

We talked with researchers and patient engagement leads in Canada and the United Kingdom, as well as a patient representative. We found three ways that could help researchers and patients find each other. One way is a case-by-case basis, where patients are often sought with experience of a health condition that is the focus of the research. The other ways involved directories where projects were posted and could be found by patients and researchers, or a third party matched patients with research projects. We found four recruitment strategies:

1. Social marketing
2. Community outreach
3. Health system
4. Partnering with other organizations (e.g., advocacy groups)

There are many influences on finding, selecting and retaining patient partners: patient characteristics, the local setting, the opportunity, work climate, education and support. We hope study results will provide a useful starting point for research teams in recruiting their patient partners.

**Abstract:**

**Background**

Patient engagement in clinical trials and other health research continues to gain momentum. While the benefits of patient engagement in research are emerging, relatively little is known about recruiting patients as research partners. The purpose of this study was to describe recruitment strategies and models of recruiting patients as partners in health research.

**Methods**

Qualitative descriptive study. Thirteen patient engagement leads and health researchers from Canada and the United Kingdom, as well as one patient representative from a national patient organization (7 female) completed semi-structured interviews.

**Results**

Recruitment infrastructures available to respondents varied, but could be categorized into three models including the traditional, third-party and directory models. Four categories of recruitment strategies were identified, representing multiple ways of recruiting patient partners: social marketing recruitment, community outreach recruitment, health system recruitment, and partnering recruitment.

**Conclusions**

Multiple recruitment strategies were identified for engaging patient partners in research, and some common factors influenced recruitment. Study findings contribute to the evidence base in patient engagement and provide guidance for research teams to help identify potential recruitment methods for their patient partners.

## Background

Patient engagement in clinical trials and other health research continues to gain momentum. Engaged patients are research *partners*, involved in the design, conduct and governance of health research [1–4]. Patient engagement can improve the relevance of health research, help transfer research findings into practice, and can ultimately improve patient outcomes [1, 4–7]. Similar improvement in health outcomes are reported when patients are engaged in their healthcare decisions [8]. Levels of patient engagement in research are possible, ranging from informing and consulting, to collaboration with researchers, to fully patient-led projects [9–11]. Patients can be engaged at all stages of the research process including prioritizing study topics, collecting or analyzing data, and knowledge translation activities [1, 2, 4, 10]. Funders of research increasingly require researchers to engage patients in their research [12].

Public involvement in research is advanced in the United Kingdom partly through the leadership of INVOLVE, a government-funded entity supporting public involvement in the National Health Service (NHS) and in health research [13]. By contrast, patient engagement in Canada and the United States has a shorter history. In 2010, the Patient-Centered Outcomes Research Institute (PCORI) was established in the United States as a main funder of comparative clinical effectiveness research that requires patient engagement in all funded studies [14, 15]. In 2011, the Canadian Institutes of Health Research (CIHR) announced Canada’s Strategy for Patient-Oriented Research (SPOR), envisioning that “*patients are active partners in health research that will lead to improved health outcomes and an enhanced health care system*” [16]. Other active areas of patient engagement include Health Technology Assessment and drug development [17].

While the benefits of patient engagement in research are emerging, the evidence is variable, and relatively little is known about its methods and impact [1, 3, 4, 18]. Recruiting patients as research partners is especially challenging, particularly for researchers new to patient engagement [4, 16]. Recent systematic reviews [4, 10] reveal that most studies use convenience sampling to identify patient partners, rather than random selection methods, and little (if any) information is provided about experiences with any recruitment method. A key gap in the literature is an understanding about the resources and strategies needed for recruitment, as well as facilitators of and barriers to, the recruitment of patient partners. Reviews suggest that patient engagement is likely feasible in many settings, but research that aims to identify methods for achieving engagement is sorely needed [4, 10, 15]. We suggest that engagement must begin by meaningfully identifying and recruiting patient partners. To that end, this research study aimed to describe strategies for recruitment and influencing factors. To our knowledge, this study is one of the first to specifically explore recruitment strategies for patient engagement in health research.

## Methods

### Study conception and patient involvement

A key outcome of Canada’s Strategy for Patient-Oriented Research (SPOR) was the creation of Support for People and Patient-Oriented Research and Trials (SUPPORT) Units across the country [19]. Units are charged with facilitating patient-engaged research on jurisdictional priorities. At the time of data collection, units across Canada were in various stages of development, but all were expected to be actively facilitating the recruitment of patient partners. We used definitions in the SPOR framework. Patients referred to “*an overarching term inclusive of individuals with personal experience of a health issue and informal caregivers, including family and friends,*” while partnering could include “*meaningful and active collaboration in governance, priority setting, conducting research and knowledge translation*” [16].

Recruitment strategies were defined as the methods participants used to find patients who would be interested in becoming a patient partner. We also looked at the infrastructures that are available to support recruitment. Recruitment infrastructures were defined as the structures or platforms available to facilitate recruitment.

Canada’s SPOR national patient engagement working group (of which HE and LV are members) identified recruiting patient partners as an important research gap, and the Newfoundland and Labrador (NL) SUPPORT Unit offered to undertake this study. The Unit is advised by a Patient Advisory Council (PAC), comprised of 21 members of the public from across the province. At the outset, the council endorsed this study’s significance and focus. A summary of the interview thematic analysis was provided to the Patient Advisory Council who were invited to comment and identify gaps in recruitment strategies from a patient perspective. Members’ feedback is incorporated into the discussion section.

### Study design

A qualitative descriptive design is a form of naturalistic inquiry making no theoretical assumptions about the data. Its goal is to present the data in the language of participants, without aiming to interpret the data in theoretical ways. The end result is a comprehensive summary of the event in question [20]. Qualitative description was a logical choice of study design as a key goal is to provide readers with a comprehensive account of recruitment strategies currently in use.

### Sampling and recruitment

Through authors’ professional networks, purposive sampling [20, 21] was used to identify patient engagement leads from SUPPORT Units across Canada. Six leads were identified representing Western, Central, and Eastern Canada. To include an international perspective, a key lead from the United Kingdom’s INVOLVE and the United States’ Patient-Centered Outcomes Research Institute (PCORI) was identified and invited to an interview. An invitation was also extended to Patients Canada, in order to include a patient perspective on recruitment strategies.

An email invitation was sent to these nine patient engagement leads to participate in the study. Leads were also invited to suggest health researchers at their sites with experience recruiting patients as partners in research. These leads identified six researchers to whom email invitations to participate were also extended. In all, 15 study invitations were sent, with only one refusal owing to time constraints during the short study duration. This purposive sampling strategy provided a broad range of perspectives on recruiting patients as partners in research, focusing in the main on the Canadian context, but also including an international lens.

### The interviews

Interviews were conducted in person or by telephone by one of the authors (DR) between June and August 2016, ranged from 30 to 60 min, were audio-recorded and transcribed verbatim. They covered several key areas: 1) specific recruitment strategies used to recruit patients as partners; 2) facilitators of, and barriers to, recruiting patients as research partners; and 3) challenges to successful recruitment. A main aim of the study was to address a key gap in the literature on patient engagement, that of specific recruitment strategies – the focus of this paper.

### Data analysis

Content analysis was used to summarize the data pertaining to recruitment strategies for patient research partners. Transcripts were read and re-read several times by one investigator (LV). Interview data were then isolated and organized around interview topics. Data pertaining to specific recruitment strategies and models were utilized to identify and index emerging categories and themes for the current paper. Two investigators then separately read and re-read the isolated data (LV, HE), and used the method of constant comparison to inductively sub-code the data relevant to recruitment strategies [20, 21]. Essentially, data were compared between and within transcripts to establish analytical categories, with a constant shifting back and forth among transcripts to compare the perceptions and experiences of participants [20, 21]. When both investigators had completed their separate analyses, they discussed categories and themes. Agreement was over 90%, and the analysis was then presented to the broader research team and the patient advisory council for peer review. We asked the council to provide a patient perspective on the recruitment framework and identify whether there were any gaps in the recruitment strategies.

#### Reflexivity considerations

As researchers inherently supportive of patient engagement, we did our best to be neutral in the conduct of interviews and during data analysis. We assumed that all participants would be supportive of patient partners in research, and challenged ourselves to actively listen for exceptions. To help balance researcher perspectives (including our own), we invited our patient advisory council for their feedback, which many members keenly provided.

## Results

A total of 15 participants were invited to participate, of which 14 agreed to be interviewed (7 female). All interviewees were involved in recruiting patient partners for health research. A description of the sample is shown in Table 1.

**Table 1:** Role and region of respondents

Role	Number	Country
Patient engagement lead SUPPORT Unit	6	Canada
Public involvement advisor/manager	2	United Kingdom
Clinical investigator	3	Canada and United Kingdom
Non-clinical investigator	2	Canada and United Kingdom
Patient partner in health research	1	Canada

Results are organized around three key themes that arose from interviews. Namely, 1) Infrastructure used for recruiting patient partners; 2) Specific strategies for recruitment; and 3) Factors that influence recruiting patients as partners.

### Recruitment infrastructure

Recruitment infrastructures varied, but could be categorized into three models:

1. Traditional model: this model focuses on a case-by-case recruitment approach and is very much driven by the subject of research itself. Recruitment starts when a project team is looking for patient partners. Recruitment support might be available in various ways, but no formal structures (such as directories) are available.
2. Third party model*:* this model is similar to a matching service. Generally, a third-party with access to patient directories helps researchers and patients find the right match. Recruitment could start before a specific engagement opportunity becomes available. A third-party provides assistance and can search through the directory to match patients and researchers.
3. Directory model: this model is similar to a dating service. The model focuses on the creation of an (often) online directory of patients who are interested in partnering with researchers. A key difference between this model and the third-party model is that access is generally not controlled. Researchers can post opportunities for engagement, while patients can search for opportunities and can directly apply for research projects.

Choice of model appeared contingent on local preferences and resources. Some interviewees preferred to work on a case-by-case basis (‘traditional model’) and noted that recruiting is driven by the subject of the research and the patient population on which it is focussing. One of the advantages of this model is that patients can be directly connected to a project.

*“I’m not looking to recruit just anybody because I want them to be tied to meaningful projects.... I am not developing a big roster of patients and not knowing where to connect them”* (Patient engagement lead, Canada).

Other interviewees described a ‘third party model’. This third party was either initiated by a Faculty of Medicine, a provincial government entity or a SUPPORT Unit. In Western Canada, for example, a network was initiated by a Ministry of Health to include patients, families and caregivers with an interest in health research. The Patient Voices Network was originally designed to match patients with health care providers running quality improvement initiatives. Currently, the network is expanding its mandate to include recruiting of patients for involvement in research:

“*Volunteers are people who have been recruited and have gone through an orientation that gives them sort of a basic background to what Patient Voices Network is, what type of engagement they can expect to hear about, a little about the health system overall and a segment on how do we work together....”* (Patient engagement lead, Canada).

Some interviewees described a directory model. One SUPPORT Unit created an online registry where patients could connect with research teams. In the United Kingdom, the National Institute of Health Research developed an online system called People in Research. This system is increasingly popular among researchers and the public for finding interesting opportunities. A reported advantage of this model is that it saves researchers time and stress.

*“It takes a lot of the stress out of it for other researchers in that they have a big pool of people here which they can use as a recruiting ground rather than having to go out themselves”*(Public involvement advisor/manager, United Kingdom).

### Recruitment strategies

A diverse range of recruitment strategies for patient engagement was identified by participants. They were categorized as follows:

1. Social marketing recruitment: this includes methods such as advertisements on radio, TV, newspapers, social media and public spaces such as churches, schools, libraries and waiting rooms.
2. Community outreach recruitment: this includes methods such as town hall meetings, contact with community leaders, booths or presentations at community events, fairs and festivals.
3. Health system recruitment: this includes recruitment by health care providers or research staff who approach potential patients within the health care system.
4. Partnering recruitment: this includes recruitment in collaboration with an organization or group who have members or represent a particular patient perspective such as advocacy groups or charitable organizations. Another partnering strategy could be partnering with a marketing company who have panel members.

A mix of strategies was commonly reported. Strategies varied depending on the research topic, team expertise, required perspectives, experiences and skills. Each strategy can be used within each infrastructure, however the recruitment process and approach might be slightly different. In the third-party model and directory model patients are often recruited based on their interest in becoming involved in health research rather than an interest in a specific research project.

#### Social marketing recruitment

Interviewees mentioned ‘limited required relationships’ and ‘little labor efforts’ as advantages of social marketing. They also noted disadvantages such as the costs of radio, TV and newspaper advertisements. While a large number of people could easily be reached with social marketing, only a small proportion of those would actually be interested in partnering for health research. Interviewees were aware that patients had to self-identify as potential partners, and so the strategy is most likely to recruit those who have the time and resources to become involved (e.g., retirees). Nonetheless, this strategy could be successful:

*“We had one specific project that was very general in the sense of the patient population that we were looking for. I think we had somewhere around 45 people who applied for one position just off [website]”* (Patient engagement lead, Canada).

#### Community outreach recruitment

An advantage of outreach recruitment was that efforts could be targeted to a specific community or group such as minority or cultural groups. Time and resources were necessary for this method:

“*There are a number of organizations that are going out to community events especially ones where there are some of the minority ethnic communities.... going along to those events and having a stand there to talk about various issues whether it’s about diabetes in the community or mental health issues....That is appreciated far more than if we were having those discussions in a health clinic or within a research facility....going out to the public has worked for a lot of the organizations very well”* (Public involvement advisor/manager, United Kingdom).

#### Health system recruitment

Health system recruitment depended heavily on existing relationships. Thus, the strategy can be quite successful for (clinical) researchers who have personal contacts with patients, either in clinical settings or from previous research:

*“I think that this is a challenge for people who don’t have a clinical practice associated with the type of work that they do. The best way that I have recruited patients to be part of my project; either people who have participated in one of my previous research projects and we had developed sort of a friendly relationship with them or they had expressed interest in the research itself”* (Clinical investigator, Canada).

Here, the implication is that whether a potential partner is known to a researcher through previously being a research participant, or because he or she is a patient of a clinician-researcher, recruitment will be less challenging.

#### Partnering recruitment

Interviewees noted that partnering with patient organizations requires well-established relationships. One interviewee recalled developing a database of patient groups and organizations from which to begin establishing relationships. However, the response to invitations to partner with these groups was initially limited.

*“We invited them to receptions but the response hasn’t been as good as we would like so we decided to get down and be more relationship focused and actually go and meet with individuals, so go to their meetings, and pitch what we are doing and try to connect that way”* (Patient engagement lead, Canada).

A non-clinical investigator partnered with a marketing company to engage the public in heath policy and ethical questions. This method was quite successful to randomly recruit members of the public.

“*It is very difficult to randomly recruit members of the public.... So what we have gone back to using was the panels that are put together by companies who do public surveys and marketing. Those panels are typically hundreds of thousands of people that have agreed for one form of research or another”* (Non-clinical investigator, Canada).

Another team partnered with an umbrella organization for smaller advocacy groups or support groups for rare conditions:

*“We had those individual patients who talked very much about their own experiences, but then we had the advocacy groups where they could talk to some general issues that people in that group tend to face”* (Clinical investigator, Canada).

### Influential factors on the recruitment of patient partners

**Fig. 1:**
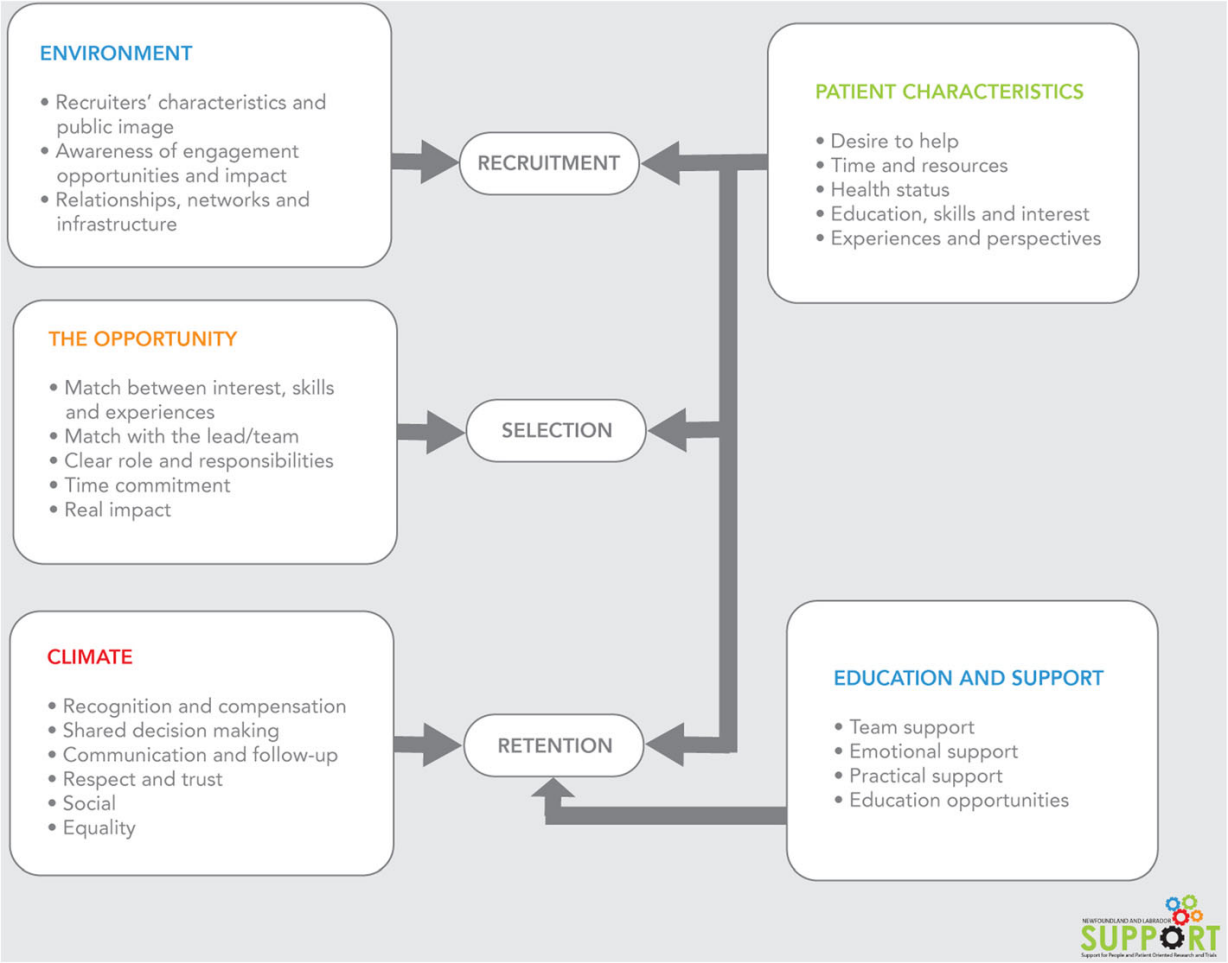
Conceptual framework for recruiting patient partners. (figure modified from the Child Welfare Information Gateway)

Numerous factors were identified by the interviewees as influential for the recruitment, selection and retention of patient partners. The factors were categorized into five main themes and visualized in a conceptual framework (Fig. 1).

Influential factors suggest the benefits of establishing meaningful relationships, networks and infrastructure facilities. Respondents reported the importance of selecting recruitment strategies that fit with the patient characteristics. It was widely emphasised that a clear description of the role, responsibilities, commitment and (potential) impact was helpful to recruit and select patients. Communication, recognition and compensation were emphasised as key factors for retention. Table 2 summarizes main themes identified from responses.

**Table 2:** Summary of main factors influencing recruitment

Main factors	Factor description and representative quotes
Environment • Recruiters’ characteristics and public image • Awareness of engagement opportunities and impact • Relationships, networks and infrastructure	The need for an environment in which the public has an awareness about engagement opportunities and the (potential) impact of patient engagement was emphasized. Furthermore, relationships, networks and infrastructure facilities such as directories could increase the success of recruitment. The recruiters’ characteristics and their public image were also noted as influential factors. Interviewees widely emphasized that the recruitment strategy should fit with the patient characteristics the team is hoping to recruit. *“One of the biggest challenges for us is the awareness of what we are trying to accomplish in the public. Typically when patients hear research recruitment they think about ´we want to recruit you so we can study you" (Patient engagement lead, Canada).*
Patient characteristics • Desire to help • Time and resources • Health status • Education, skills and interest • Experiences and perspectives	It was reported that patients who have time and an interest in the research topic were more likely to become engaged. It was also emphasised that patients bring skills, perspectives and experiences. Drop-out reasons were reported such as health issues or caregiving responsibilities, different priorities, frustration with the pace of the project and an overload of work or volunteer activities. For example, one respondent described their strategy as follows, *“....some people, especially people with mental health problems will drop out last minute and you have very little notice to deal with that. We often recruit perhaps more than you anticipate; you need to cover that scenario”* (Public involvement advisor/manager, United Kingdom).
The opportunity • Match between interest, skills and experiences • Match with the lead/team • Clear role and responsibilities • Time commitment • Real impact	It was widely reported that a clear description of the role, responsibilities, commitment and (potential) impact was helpful to recruit and select patients. For example, one respondent commented, *“.....a key thing is about clarity. Clarity in terms of roles and responsibilities and how much people will be involved as well, I think that’s really important.”* (Public Involvement advisor/manager, United Kingdom). The importance of thinking about the perspectives, skills and experiences needed for the research project was frequently mentioned: *“You want to have patients on your project that would complement your team. When I am thinking about a project, I’m thinking about researchers that I want. .... maybe I need a biostatistician ....maybe an oncologist... So I think that I would have the same approach to finding patient advisors”* (Clinical Investigator, Canada). An initial interview was recommended to ensure a good fit with the project and the team. It was also suggested to start thinking about recruitment in very early stages as it takes time to recruit and select patient partners.
Climate • Recognition and compensation • Shared decision making • Communication and follow-up • Respect and trust • Social • Equality	Recognition and compensation were emphasised as key factors for retention. Interviewees covered expenses such parking fees and travel costs. Multiple interviewees offered financial compensation such as an honorarium, hourly rate, per diem compensation or gift cards. Compensation varies among projects, provinces within Canada and in other countries. *“You have to find out what is important to the patients themselves, in terms of how to be recognized and compensated for what they are doing”*(Patient engagement lead, Canada) Good communication was reported as another key element for retention. *“Some of the things that help with retention is good communications, consistent communications and relatively frequent communications - not going 3–6 months with no contact and then expecting them to just pick it up”* (Patient partner, Canada). Interviewees also emphasised the importance of social aspects such as providing drinks and decent food. Respect, equality and trust were widely mentioned as essential for retention. The level of engagement was also mentioned by interviewees, with shared decision-making and co-production noted as important.
Education and support • Team support • Emotional support • Practical support • Education opportunities	Education opportunities, ongoing mentorship and support were reported as influential factors for retention. A number of emotional and practical considerations have to be taken into account while working with patient partners such as supportable furniture, timely breaks, transport facilitation and accessible accommodation. For example, one respondent mentioned: *“...the other thing that needs to be paid careful attention to on the food side is some patients have very strict dietary requirements and they need to eat at particular times otherwise they are going to be in a very difficult situation with their health. Those are all considerations that need to be taken into account”* (Patient partner, Canada).

### Challenges

Multiple challenges were identified in recruiting patient partners. Obvious barriers included time for relationship building and (financial) resources. Frequently mentioned was also the lack of public awareness about the need and (potential) impact of patient engagement, as recruitment is typically related to subjects of research.

“*It is just foreign to most people and not just patients. You have to have a clear 30 second elevator pitch or email or phone call that clearly makes the distinction and allows it to see the value of why you are doing it, that is the biggest challenge in my opinion right now across the country”* (Patient engagement lead, Canada).

Multiple interviewees mentioned the struggle with recruiting a diversity of patients, as retirement-age women were the usual partners recruited. Youth and minority groups were particularly hard to recruit. Interviewees recognized the difficulty of representativeness in patient partners and suggested other team members are not held to the same standard of representativeness:

*“... you can’t assume that the economist that’s sitting at the table is representative of all the other economists or that the epidemiologist or biostatistician or clinician whatever. So I think sometimes we are a bit harder on the patients, we probably put them under a bit more pressure to be super representative and we don’t do that with the other research partners in the group”* (Patient engagement lead, Canada).

*“We try to encourage people to try and think of evening meetings, weekend meetings, doing things in other ways, having online meeting where people might be able to contribute during those outside daytime areas, just thinking outside the box slightly so we can include those other communities”* (Public involvement advisor/manager, United Kingdom).

Other challenges were related to ethics approval of research projects. For example, one interviewee noted that the local ethics authority was unclear whether patient engagement required ethical approval and if patient partners needed to complete training. Overall, interviewees noted that researchers have limited knowledge on how to recruit and engage patients as partners.

### The exception

Negative case analysis [21] revealed one respondent who did not refer to patients as partners, but rather research subjects.

“*So we are involving patients to take part in our research…to recruit patients is quite a process. You have to start off and get ethical approval to do the study in general practice, then to approach the practices, then you have to get the practices to recruit on your behalf…then as a researcher, we can contact the patients to take part in the research”* (Clinical researcher, United Kingdom).

This respondent identified many of the themes raised earlier (e.g., the importance of good communication with patients, the time needed upfront to create buy-in for the project), but these were largely in reference to patients as participants, not research partners.

## Discussion

This study found a diverse array of recruitment strategies for patient engagement that were categorized into four strategies: social marketing, community outreach, health system recruitment and partnering recruitment. Health system recruitment seems to be effective for finding patients with a particular condition or experience, while community outreach seems to be more helpful to reach specific communities such as minority or cultural groups. Social marketing tends to be successful for large groups to recruit those who are able to self-identify themselves as potential partners (often retirees, females). Partnering with marketing companies can be successful to randomly recruit the wider public, while partnering with advocacy groups or charitable organizations can be successful to reach specific perspectives on issues patients in that group tend to face.

Most of the literature on recruitment is in the context of patients as research *subjects*, though similar recruitment strategies are used in that context (e.g., [22]). The few studies that engaged patients and described their recruitment strategies [23–25], as well as recent systematic reviews [4, 26] reported similar strategies to those recounted in our interviews, most of which were forms of convenience sampling. Our patient advisory council identified a role for patient partners to help recruit additional partners, which was not raised widely in our interviews. This suggestion is similar to a recent systematic review on patient engagement in the context of research on rare diseases [26], which reported that very few studies addressed the role of engagement in knowledge translation and dissemination.

Locock and colleagues [12] encourage researchers to reflect on notions of power in the health research community, specifically how power imbalances between patients and researchers can undermine patient involvement in research. We note that power imbalances could be especially relevant in health system recruitment models, where clinician-researchers recruit patient partners from their practices. Some of our participants noted efforts to visit communities or hold meetings outside of daytime hours or in online formats that could allow more people to contribute. These efforts suggest a desire to be inclusive and are promising; however, particularly within traditional models of recruitment from clinical practices, reflections on power would be useful.

The results of this study revealed numerous influential factors on the recruitment, selection and retention of patient partners. Respondents and our patient advisory council suggested these three processes were interrelated and teams should think beyond recruitment. The conceptual framework is meant to give research teams an idea which factors they might consider for recruiting and retaining patient partners. Our findings are generally consistent with existing literature on influential factors for patient engagement [1, 14, 25, 26]. Forsythe et al. identified early contributors and lessons learned in the 50 Pilot Projects funded by the PCORI [14]. They identified communication and shared leadership strategies as “critically important” facilitators. Patients’ motivations and interest for being involved in research also impact recruitment [27]: patients are typically motivated by their individual needs and are most interested in research specific to their own condition. To that end, patient organizations have been used successfully to identify patient partners in rare disease research [26].

Our patient advisory council endorsed the framework presented herein, specifically identifying the need for clear communication upfront about time commitment, roles and expectations as keys to successful recruitment. Similarly, they endorsed the importance of the match between project and patient partner to ensure a successful partnership. Some members also commented that age and gender could influence who might respond to a patient partner request. They recognized that older, retired women are typical volunteers, but younger patient partners might be recruited for pediatric research for example. Factors that they felt could be added to the framework were: patients’ desire to learn, career growth opportunities as benefit from engagement activities, travel time and expenses required and clear expectations from both sides (patient and researcher). They also highlighted the importance of meaningful, rather than merely token ways, of engagement. They suggested that the same recruitment principles for the recruitment of key employees should be considered when recruiting patient partners. The importance of good communication techniques and design was highlighted as another important factor. The ‘Keep it simple’ method and working together with marketing and design/art experts was advised by our patient advisory council.

One challenge is low public awareness about the need and (potential) impact of patient engagement, as recruitment is typically related to subjects of research. A recent study found only moderate levels of interest among patients and low levels of awareness of patient engagement [28]. It is also worth noting that even in this very aware sample, one respondent referred to recruitment of research participants, rather than patients as partners. As our council recommended, current patient partners can certainly help raise public awareness and share their experiences with potential patient partners. Our council members noted ‘competing opportunities’ as more and more researchers and health care organizations are looking for patient partners, thus risking patient partner burnout.

Study findings were based on a small set of qualitative interviews and cannot be generalized to all settings. It is encouraging, however, that similar findings were found in both Canadian and UK interviews, and factors identified as influential for patient engagement correspond to other literature. However, given the patient advisory council raised other issues related to recruiting patients as partners that were not raised widely in the interviews, it is possible data saturation was not obtained, restricting the conclusions to be drawn from the data. We note that similar themes arose after 8–9 interviews, with few new themes emerging in subsequent interviews. Thus, it is possible that patients’ and researchers’ perspectives on recruiting patients as partners raise different themes, but we have too few data to draw firm conclusions. Patients as research partners is still a relatively new area, with many choices dependent on the local context and specific research projects, making it difficult to categorize common elements and provide recommendations on best practices. Additionally, results provide researchers with several recruiting strategies without any indication of their effectiveness. Other areas for future research were identified by our patient advisory council after reviewing study findings. They suggested that the recruitment of patient partners was only the first step; while not the primary focus of this study, equally important is retention. We would add the value of research that explicitly compares patient and researcher perspectives on recruitment in order to elucidate a full range of strategies.

## Conclusions

Multiple recruitment strategies were identified for patients as partners in research, and some common factors were noted to influence such recruitment. Future research is needed to explore the effectiveness and costs of particular strategies, and how best to promote awareness of patient partners in health research. Currently, we hope study findings will provide a useful starting point for research teams in identifying potential recruitment methods for their patient partners.

What this paper adds:

What is known

- Little research specifically describes strategies for recruiting patients as partners in health research
- Recruiting patient partners is a key challenge identified by health researchers

What this study adds

- Multiple recruitment strategies can be used to recruit patient partners, including community outreach, health system, social marketing and partnering approaches
- This study provides valuable guidance for researchers and others recruiting patients as research partners and adds to the evidence base on methods of patient engagement

## Abbreviations

CIHR: Canadian Institutes of Health Research
DR: Devonne Ryan
HE: Holly Etchegary
LV: Lidewij Eva Vat
NHS: National Health Service
NL: Newfoundland and Labrador
PAC: Patient Advisory Council
PCORI: Patient-Centered Outcomes Research Institute
SPOR: Strategy for Patient-Oriented Research
SUPPORT Unit: Support for People and Patient-Oriented Research and Trials Units
UK: United Kingdom
